# Utilization of a two-material decomposition from a single-source, dual-energy CT in acute traumatic vertebral fractures

**DOI:** 10.3389/fradi.2023.1187449

**Published:** 2023-09-21

**Authors:** Patrick Tivnan, Artem Kaliaev, Stephan W. Anderson, Christina A. LeBedis, Baojun Li, V. Carlota Andreu-Arasa

**Affiliations:** Department of Radiology, Boston University School of Medicine, Boston, MA, United States

**Keywords:** fracture, bone marrow edema, dual-energy computed tomography, magnetic resonance imaging, calcium, fat density, water density

## Abstract

**Purpose:**

The purpose of this study is to utilize a two-material decomposition to quantify bone marrow edema on a dual-energy computed tomography (DECT) scanner at the cervical, thoracic, and lumbar spine acute fractures in correlation with short tau inversion recovery (STIR) hyperintensity on magnetic resonance imaging (MRI) in comparison with the normal bone marrow.

**Materials and methods:**

This retrospective institutional review board–approved study gathered patients over 18 years old who had acute cervical, thoracic, or lumbar spinal fractures scanned on a DECT scanner. Those who had a spinal MRI done with bone marrow STIR hyperintensity within 3 weeks of the DECT were included. The water (calcium) and fat (calcium) density (mg/cm^3^) measurements of the region of interest of the bone marrow were obtained at a normal anatomic equivalent site and at the fracture site where STIR hyperintensity was noted on MRI. A statistical analysis was performed using the paired *t*-test and Wilcoxon signed rank test (*p* > 0.05).

**Results:**

A total of 20 patients met the inclusion criteria (males *n* = 17 males, females *n* = 3). A total of 32 fractures were analyzed: 19 cervical and 13 thoracolumbar. There were statistically significant differences in the water (43 ± 24 mg/cm^3^) and fat (36 ± 31 mg/cm^3^) density (mg/cm^3^) at the acute thoracic and lumbar spine fractures in correlation with edema on STIR images (both paired *t*-test <0.001, both Wilcoxon signed ranked test *p* < 0.01). There were no significant differences in the water (−10 ± 46 mg/cm^3^) or fat (+7 ± 50 mg/cm^3^) density (mg/cm^3^) at the cervical spine fractures.

**Conclusion:**

The DECT two-material decomposition using water (calcium) and fat (calcium) analyses has the ability to quantify a bone marrow edema at the acute fracture site in the thoracic and lumbar spine.

## Introduction

Traumatic spinal fractures are a concerning injury that can occur from a variety of mechanisms including high-energy falls and motor vehicle accidents (1, 2). In the United States, approximately 1 million trauma cases occur yearly, and cervical fractures are seen in approximately 6% or 58,000 cases per year (2). These fractures can cause significant morbidity and/or mortality annually and incur a significant economic burden (3, 4).

Early detection of acute spinal fractures in the emergency setting allows for the appropriate management of these injuries. For at least the recent decade, the multidetector computed tomography (MDCT) has been shown to be a reliable method to assess for spinal fractures (5–10). Specific spinal reformats with 1–3 mm slices, bone algorithms, and sagittal and coronal reformats can improve the sensitivity for these fractures (11).

While conventional CT imaging can detect the evidence of acute fractures in most scenarios, the evaluation can be challenging when trying to determine subtle fractures or the age of a fracture (acute vs. chronic). Magnetic resonance imaging (MRI) has often been utilized in these circumstances due to its ability to detect bone marrow edema (BME), a finding that may be seen with acute fractures (12, 13). MRI, however, is often not sufficient alone for the evaluation of the spine due to inferior detail of the osseous trabecula and cortices provided with the addition of the higher cost, prolonged time of exam, and poor accessibility.

In recent years, dual-energy computed tomography (DECT) has been developed to provide additional details when obtaining a conventional CT. On DECT, a patient is scanned with two different photon energy regimes with relative attenuation being measured at the different photon energy levels. The relative differences in attenuation that occurs between materials in specific voxels at different energy levels can allow for material composition curves to be generated (14). Extrapolating these compositional differences into computational images can allow for the detection of acute fractures through the detection of an increased water content, a sign of a bone marrow edema, and correlate to increased short tau inversion recovery (STIR) signal on MRI (12, 15–17).

In this study, we sought to evaluate whether DECT, specifically the two-material decomposition analyzing fat and water content after the subtraction of calcium attenuation on the Gemstone Spectral Imaging (GSI) on the GE Revolution (General Electric Healthcare, Chicago, IL, USA), had utility in the detection of acute traumatic spinal fracture in a different context than many prior studies, namely, a Level 1 trauma center, as detection and differentiation of acute from chronic spinal fractures is especially important in this setting.

## Methods

### Ethics approval and consent

This retrospective study was approved by the institutional review board (IRB) and was HIPAA compliant. Informed consent was waived.

### Patients

**Figure 1:**
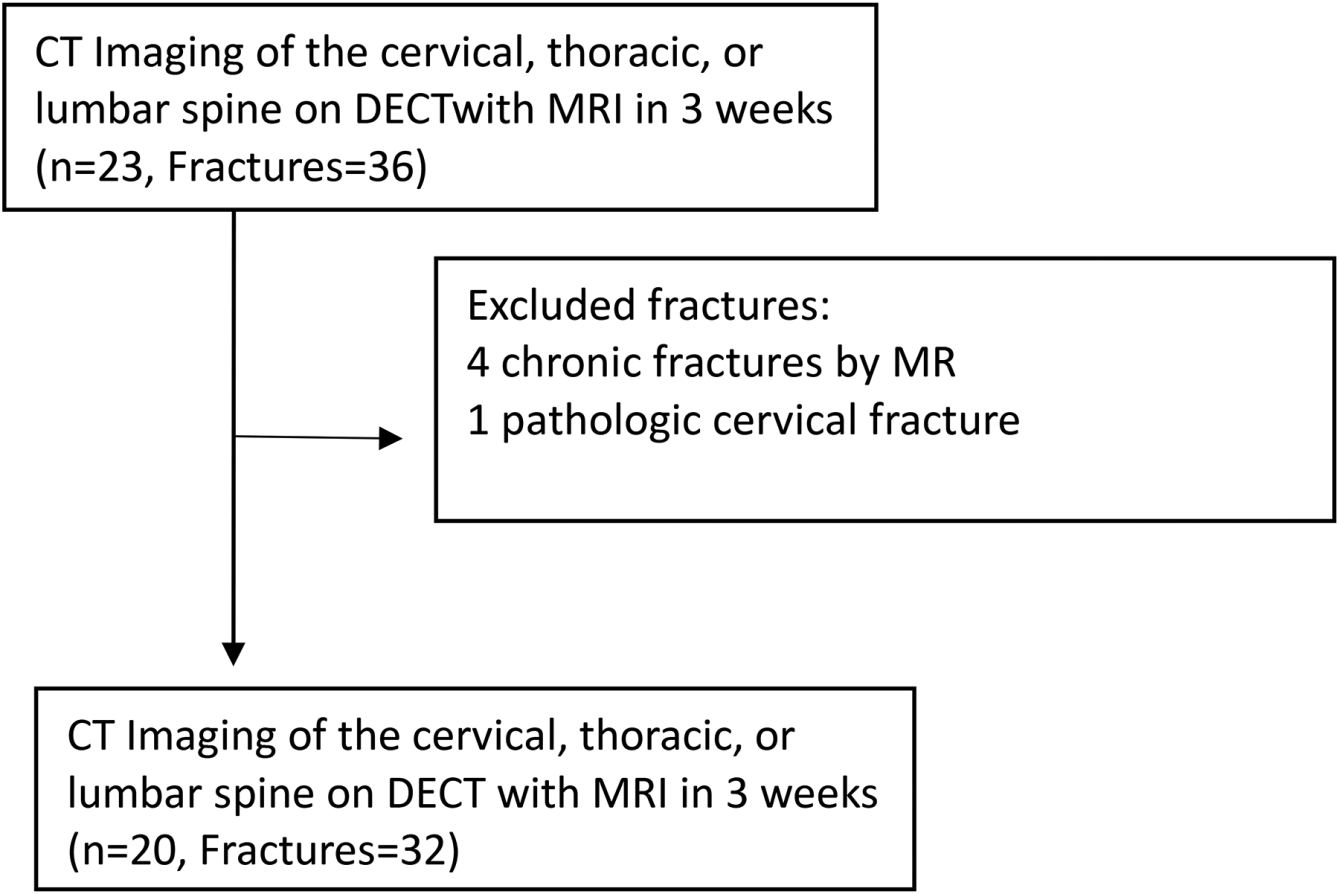
Patient selection flowchart.

We retrospectively included patients 18 years or older who consecutively presented to our Emergency Department (ED) between 1 August 2019 and 28 February 2020 in the setting of substantial trauma to the cervical spine or to the chest, abdomen, or pelvis, including high-energy blunt and penetrating injuries. Those that received admission trauma scans on the Revolution DECT platform were selected for inclusion. Of this cohort, those who underwent spine MRI within a 3-week period from the initial DECT demonstrating BME on STIR sequence were included in this study (Figure 1). Three weeks was considered a reasonable short period to correlate between bone marrow edema in DECT and STIR sequence.

Patients under 18 years old or those who had a spinal MRI done after a 3-week period after the acute trauma were excluded.

### Imaging

All patients underwent imaging on the GE Revolution single-source, DECT 256-detector row scanner (General Electric Healthcare, Chicago, IL, USA), that allows for fast kV switching during a dual-energy data acquisition. GSI is standardly turned on in our GE Revolution during our trauma CT examinations with the recommendation for the technicians to turn off the GSI if the patient exceeds 280 lb in weight. For each DECT, the kV utilized were 80 and 140. Iodinated contrast—iopamidol (Isovue 370, Bracco, Princeton, NJ, USA)—was used for any of the below-described contrast-enhanced studies. The appropriate tube current was automatically determined using the mA assist feature (noise index = 18.4 for CT thoracic and lumbar spine; noise index = 6.2 for CT cervical spine; noise index = 15.4 for CT chest; noise index = 12.3 for CT abdomen and pelvis).

Contrast-enhanced CT chest, abdomen, and pelvis imaging was performed using our trauma protocol that employs 100 ml of iodinated contrast and full helical scanning at 1.25 mm slice thickness/intervals. Imaging is performed in the chest during a 30 s arterial phase delay and in the abdomen and pelvis with 70 s delay.

Reconstructions in the sagittal and coronal planes were standardly generated. Additional delayed images are often obtained at the discretion of the radiologist if there was an evidence of pelvic or solid organ lesion, but these were not utilized in this study.

A dedicated non-contrast imaging of the cervical, thoracic, and lumbar spine was performed with helical imaging at 1.25 mm slice thickness/intervals. Imaging was reviewed in the soft tissue and bone algorithm reformats with 2 mm thickness/interval coronal and sagittal reconstructions.

CT angiograms of the neck were performed extending from the skull base to the aortic arch with 1.25 mm slice thickness/intervals after the intravenous (IV) administration of 100 ml of Isovue 370 (iopamidol) using a smart prep formula assessing for IV contrast within the aortic arch. Imaging was reviewed in soft tissue and bone algorithm reformats with 2 mm thickness/interval coronal and sagittal reconstructions.

For MRI imaging, both MRI imaging at 1.5 and 3 T scanners of the relevant spinal area performed at our institution and on occasion at outside institutions (before transfer) were included. These imaging sequences included sagittal STIR and T2 and T1 sequences, in addition to axial T1 and T2 sequences at a minimum. Correlation of the bone marrow edema was made with sagittal STIR sequence (TR:2500, TE:90 on 1.5 T Phillips, Achieva scanner and TR:3782, TE:75 on 3 T Phillips Ingenia scanner).

### Target and reference vertebrae

Ellipsoid regions of interest (ROIs) were placed by a board-certified radiologist (with 2 years of faculty-level experience) at the site of the fracture where STIR hyperintensity was demonstrated on MRI and at the anatomic equivalent site. ROIs of arbitrary sizes were placed within the medullary bone in the expected region of the bone marrow edema and were drawn to exclude the adjacent cortical bone. Sagittal CT reformatted images (1.5 mm thickness for the cervical spine and 2.5 mm thickness for the thoracic and lumbar spine) were used for ROI placement. Fractures of the vertebral body and posterior elements of those vertebrae were included. Occipital condyle fractures were included as examples of cervical spine fractures.

Water (calcium) and fat (calcium) ROI measurements (mg/cm^3^) for quantitative analysis of the water and fat density in the bone marrow at the fracture site were performed on the GE AW Server 3.2 Ext 2.0.

### Data analysis

A statistical analysis was performed using the paired *t-*test to compare the measurements at the fracture site and a normal anatomic site in the same patient. Wilcoxon signed rank test was also performed after the *q*-plot analysis demonstrated lack of normal distribution for data sets. Two tailed *p-*values less than 0.05 were considered statistically significant.

## Results

### Patient cohort

A total of 32 fractures from 20 patients were identified with patient characteristics and fracture location listed in Table 1. The cohort was 85% male (17/20) and 15% female (3/20) with an age range of 20–88 years (mean of 58.8 years). The mean time elapsed between CT and MRI was 1.9 days.

**Table 1:** Patient and fracture characteristics.

Cohort (*n* = 20)	
Age (years ± SD)	58.8 ± 22.6
Male	17/20 (85%)
Female	3/20 (15%)
Fractures (*n* = 32)	
Cervical	19 (59%)
Thoracic	8 (25%)
Lumbar	5 (16%)

### Target vertebrae

A total of 32 acute fractures were detected on DECT with corresponding STIR hyperintensity on MRI. Of these fractures, 59% (19/32) occurred in the cervical spine, 25% (8/32) in the thoracic spine, and 16% (5/32) in the lumbar spine. The cervical fractures included fracture dislocation, fractures of vertebral bodies or lateral masses (11/19), C1 ring fractures (2/19), odontoid (1/19), posterior elements (4/19), and one fracture of the occipital condyles adjacent to a C1 fracture (1/19). In one case at C6, both laminae were fractured, and these were measured as two separate cervical fractures as they appeared distinct by both MRI and CT imaging.

In the lumbar and thoracic spine, 13/13 fractures were of the vertebral body with 11 compression fractures, one burst fracture, and one oblique vertebral fracture.

Four additional fractures involving the vertebral body were excluded as they did not demonstrate STIR hyperintensity, which is present in most acute fractures (18). An additional vertebral body fracture was determined to be pathologic in the context of cervical vertebral metastases and was also excluded.

### Acquisition of CT images

Two of the 13 (2/13) thoracic and lumbar and spine DECT were done without contrast with our lumbar spine protocol as per above. The remaining 11/13 lumbar and thoracic spine fractures were detected on CT performed with iodinated contrast according to our standard trauma protocol as listed above.

Five out of the 19 (5/19) cervical spine fractures were noted on CT angiograms of the neck. The remaining fractures (14/19) were noted on non-contrast studies of the cervical spine.

### Image analysis

**Figure 2:**
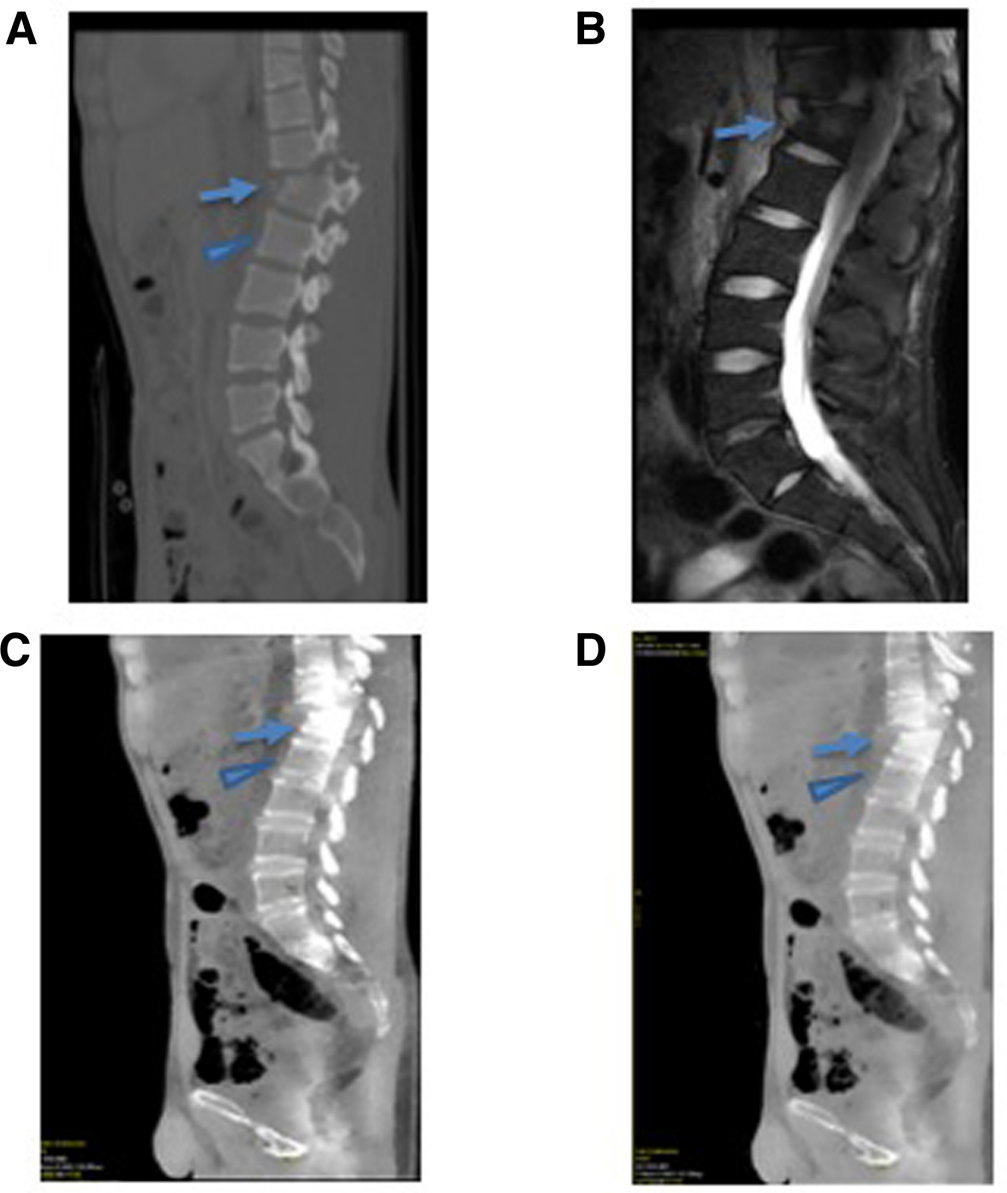
A 25-year-old male presenting with an acute thoracic spine fracture after a fall from a tree based on GSI dual-energy CT analysis with MRI correlate. (**A**) The conventional CT scan of the thoracic spine demonstrates a fractured T12 (arrow) and uninvolved L1 vertebral body (arrowhead). (**B**) The MRI STIR imaging demonstrates an increased signal within the fractured vertebral body (arrow) without a signal abnormality in the uninvolved adjacent L1 vertebral body (arrowhead). (**C**) Water (calcium) and (**D**) fat (calcium) images demonstrated a quantifiably increase in water content at the fractured T12 vertebral body (arrow) compared with the normal L1 vertebral body (arrowhead).

**Figure 3:**
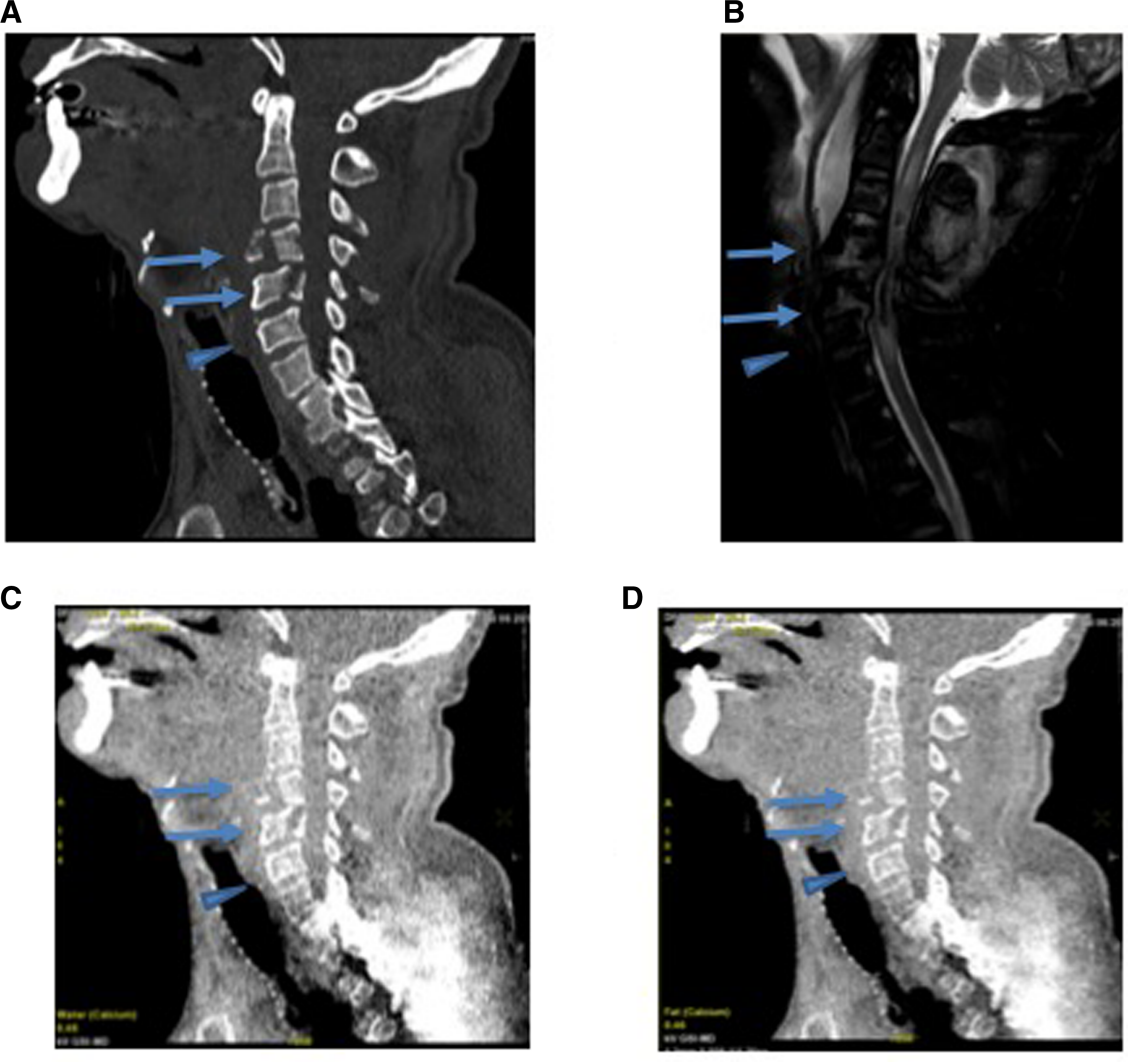
A 57-year-old male presenting with an acute cervical fracture after a 50-ft fall based on GSI dual-energy CT analysis of with MRI correlate. (**A**) The conventional CT scan of the cervical spine demonstrates fractures of C4 and C5 vertebral bodies (arrow) and adjacent uninvolved C6 vertebral body (arrowhead). (**B**) The STIR imaging of the cervical spine demonstrates an increased signal in the region of the fractures (arrows) without signal abnormality in the uninvolved C6 (arrowhead). (**C**) Water (calcium) and (**D**) fat (calcium) images show no significant change in water content between the fractured C4 and C5 (arrow) and normal C6 (arrowhead).

Elliptical ROIs were placed in the marrow cavity in regions of the fracture that demonstrated an increased STIR signal on the corresponding MRI. These ROIs were carefully placed avoiding the cortex, which was particularly challenging in the cervical vertebra, given the small size of the marrow cavity. For the normal, non-fractured control bone, a similar approach was utilized to avoid the corticated bone (Figures 2, 3).

### Lumbar and thoracic spine

The average difference between the thoracic and lumbar spinal fractures was +43 ± 24 mg/cm^3^ for water density (mg/cm^3^) on water (calcium) analysis and 36 ± 31 mg/cm^3^ for fat density on fat (calcium) analysis.

There were statistically significant differences in the bone marrow water density (mg/cm^3^) with water (calcium) at the acute fractures of the thoracic and lumbar spine with respect to the normal bone in correlation with edema noted on STIR (paired *t*-test *p *= 4.8 × 10^−5^ = 0.000048, Wilcoxon signed rank test *p* = 1.47 × 10^−3^ = 0.0147) (Table 2).

**Table 2:** Thoracic and lumbar spine fractures.

Fracture level		Water^a^ (calcium)		Difference	Fat^b^ (calcium)		Difference
		Normal	Fracture		Normal	Fracture	
1	T3	1,003	1,024	21	1,010	1,025	15
2	T2	1,003	1,057	54	1,010	1,030	20
3	T12	1,064	1,160	96	1,049	1,139	90
4	T8	1,032	1,059	27	1,009	1,031	22
5	T6	1,005	1,053	48	986	1,033	47
6	T12	1,001	1,049	48	973	1,006	33
7	T11	1,020	1,069	49	992	1,056	64
8	T12	1,020	1,112	92	992	1,083	91
9	L1	1,020	1,045	25	992	999	7
10	L2	1,020	1,049	29	992	1,000	8
11	L4	1,020	1,046	26	992	984	−8
12	L3	1,018	1,051	33	985	1,027	42
13	L1	1,021	1,038	17	995	1,035	40
Thoracic/lumbar		1,019	1,062	43	998	1,034	36

^a^ SS (*t*-test *p* = 4.8 × 10^−5^, Wilcoxon signed rank test *p* = 1.47 × 10^−3^).

^b^ SS (*t*-test *p* = 1.0 × 10^−3^, Wilcoxon signed rank test *p *= 1.13 × 10^−3^).

Also, there were statistically significant differences in the bone marrow fat density (mg/cm^3^) with fat (calcium) at the site of acute fractures of the thoracic and lumbar spine with respect to the normal bone in correlation with hyperintensity noted on STIR (paired *t*-test *p *= 1.0 × 10^−3^ = 0.001, Wilcoxon signed rank test *p* = 1.13 × 10^−3^ = 0.00113).

### Cervical spine

The average difference between the normal and fractured bone for the cervical spine was −10 ± 46 mg/cm^3^ for water density on water (calcium) images and +7 ± 50 mg/cm^3^ for fat density on fat (calcium) images.

There were no statistically significant differences in the bone marrow water or fat density (mg/cm^3^) in correlation with STIR hyperintensity at the acute fractures in the cervical spine in comparison with non-fractured bone (paired *t*-test *p* = 0.36 and Wilcoxon signed rank test *p* = 0.40, *t*-test *p* = 0.56, Wilcoxon signed rank test *p* = 0.75, respectively) (Table 3).

**Table 3:** Cervical spine.

Fracture location		Water (calcium)^a^		Difference	Fat (calcium)^b^		Difference
Fracture		Normal	Fracture		Normal	Fracture	
1	C2	1,152	1,120	−32	1,030	1,098	68
2	C4	1,041	1,068	27	1,095	1,076	−19
3	C5	1,085	1,024	−61	1,072	1,008	−64
4	C6	1,042	1,072	30	1,011	1,054	43
5	C5	1,042	1,100	58	1,011	1,068	57
6	C5	1,057	1,081	24	1,039	1,038	−1
7	C6	1,057	1,068	11	1,039	1,047	8
8	C2	1,066	1,096	30	1,042	1,071	29
9	Odontoid	1,088	1,150	62	1,063	1,124	61
10	C1	1,026	1,031	5	1,002	1,007	5
11	C1	1,120	1,052	−68	1,098	1,034	−64
12	Occipital condyle	1,067	1,082	15	1,067	1,051	−16
13	C1	1,067	1,049	−18	1,067	1,049	−18
14	C7	1,119	1,086	−33	1,081	1,046	−35
15	C6 (R)	1,119	1,012	−107	1,081	1,012	−69
16	C6 (L)	1,119	1,096	−23	1,081	1,083	2
17	C2	1,068	1,017	−51	996	979	−17
18	C4	1,120	1,056	−64	1,059	1,093	34
19	C5	1,120	1,126	6	1,059	1,184	125
Cervical		1,083	1,073	−10	1,052	1,059	7

^a^ NS (paired *t*-test *p* = 0.36 and Wilcoxon signed rank test *p* = 0.40).

^b^ NS (*t*-test *p* = 0.56, Wilcoxon signed rank test *p* = 0.75).

## Discussion

In this study, we demonstrated that there is a statistically significant increase in water density (mg/cm^3^) noted on water (calcium) and fat (calcium) in thoracic and lumbar spinal fractures in the setting of acute trauma with correlation with STIR hyperintensity on MRI, when comparison is made with the normal bone. DECT has shown a high specificity of 96% of detecting the presence of BME in vertebral fractures, as demonstrated in a published meta-analysis study (19). Several prior prospective studies have shown the ability of DECT to detect BME in acute vertebral compression fractures that were first qualitatively radiographically evident (16, 20–22). Other authors have shown these findings in the context of retrospective reviews of vertebral compression fractures (23, 24). Pan et al. and Abbassi et al. recently demonstrated an application of GSI in assessing for BME in acute vertebral compression fractures (23) and in a variety of anatomical sites (25), respectively. Single-source DECT with rapid kV switching has shown a reasonably high sensitivity and accuracy for detecting BME in vertebral compression fractures (26), and dual-source DECT has also demonstrated an excellent diagnostic performance for the detection of vertebral BME (27) in the setting of fractures.

In this study, we evaluated the role of DECT, specifically the two-material decomposition (fat and water content after the subtraction of calcium attenuation) on the GSI, in a different clinical scenario than prior studies, in the setting of acute trauma in a Level 1 trauma center in patients who underwent CT without and/or with contrast. Many of these scans performed were not optimized specifically for the evaluation of the corresponding spine within the respective imaged body section. CT scans that occur in the trauma setting can be complicated for various reasons, including incomplete clinical history, competing injuries, and, on occasion, less than ideal scanning scenarios, for example, artifact from foreign objects or the inability to place the arms out of the field of view. In most of the scans of the thoracolumbar spine in our study, imaging of the spine occurred alongside imaging of the thorax and abdomen with many of the studies having concurrent injuries to the solid organs.

We were able to demonstrate that in the acute trauma setting, GSI can be used to quantitatively assess a bone marrow edema within traumatic fractures of the thoracolumbar spine that correlated with T2 STIR signal on subsequent MRI. A statistically significant increase in water density, a sign of a bone marrow edema, was observed in the fractured bone in relation to the normal bone in these parts of the spine as detected by GSI. This correlates with prior other studies that showed that compression fractures in the vertebral bodies of the thoracolumbar spine had demonstratable BME by DECT (16, 19, 20). In our study, in addition to anterior wedging compression fractures, we observed an increased BME in other types of traumatic spinal injuries including burst and oblique vertebral body fractures.

Of the two types of analysis that we performed by GSI that removed the calcium content, we found that both the water (calcium) and fat (calcium) analyses showed statistically significant increases in water and fat density (mg/cm^3^), respectively, between the fracture and the normal adjacent bone. Unlike the conventional CT, which expresses the ROI pixel values in the context of Hounsfield units, these analyses on DECT GSI provided a quantifiable density within a voxel of a specific substance. An increase in water density (mg/cm^3^), therefore, is explained by an increased marrow edema. However, the increase we observed in fat density (mg/cm^3^) in a fracture is not physiological as the relative density in a voxel of normally fatty marrow would be expected to decrease in the setting of acute fracture due to edema. When GSI makes compositional deconstructions, namely, removing the attenuation from calcium, what remains is likely a mixture of attenuation from the remaining dominant substances in the voxel including water and fat. These analyses attempt to detect the remaining attenuation from the other substance (water in water analysis, fat in fat analysis), but the third (or additional) unquantified substances can still have an effect. As we observed, an increased density (mg/cm^3^) of both water and fat in acute vertebral fractures, it is likely that these analyses did not accurately measure the fat density (mg/cm^3^) in the marrow, and in the case of fat analysis, these analyses were possibly also analyzing water density (mg/cm^3^). Further analysis needs to be done to evaluate if more developed material decomposition can increase the sensitivity for detecting BME such as the three-material decomposition methods. For example, Pan et al. (23) recently showed that hydroxyapatite analyses with GSI has potentially improved the sensitivity over simple calcium suppression in the setting of the two-material decomposition for BME detection on vertebral compression fractures.

In contrast to the thoracolumbar spine, fractures in the cervical spine, often of the highest concern in the emergency setting and the majority in this cohort, did not show reliable BME on GSI using water (calcium) or fat (calcium) analyses. To our knowledge, we appear to be the first to make this observation, with no studies to date showing DECT being effective for demonstrating BME in cervical fractures. This finding could be due to the reduced bone marrow cavity in the vertebrae of the cervical spine in comparison with those in the thoracolumbar spine, limiting the extent of any observable edema. In our experience, placing appropriate ROIs in cervical vertebral fractures near the region of STIR hyperintensity, but excluding cortical fragments displaced into the bone marrow cavity, was very challenging. If an ROI was taken in a region of cortical fragments, the water content was often found to be excessively increased possibly due to an incomplete calcium suppression of the cortical bone.

In addition, while the thoracolumbar spine did not pose a challenge to place the ROIs given the robust size of the bone marrow space, chronic degenerative changes in the cervical spine presented an obstacle to analysis, with endplate sclerosis in discogenic degenerative changes further reducing the bone marrow cavity. Likewise, with a calcium suppression analysis, the sclerotic, non-edematous trabeculated bone often demonstrated increased water content in relation to the normal non-edematous trabeculated bone, possibly due to an incomplete suppression. The expected observation in this context would be that the bone marrow cavity either has the same or less water content due to the increased presence of the trabeculated/sclerotic bone. Further work is needed to evaluate whether other types of GSI analyses are better for assessing BME in the cervical spine.

Interestingly, while there were both contrast and non-contrast scans in both the cervical and thoracolumbar cohorts in this study, the majority of the thoracolumbar imaging was done with contrast scans, and the majority of the cervical spine imaging was done without contrast. It is possible that the utilization of iodinated contrast accentuates the bone marrow edema with hyperemia being more evident in iodinated contrast images, thereby accounting for why our thoracolumbar fractures showed a significant BME. However, most prior studies demonstrating BME by DECT with compression fracture in the thoracolumbar spine were performed without contrast, suggesting that iodinated contrast either does not hinder or is not needed for this observation to occur (19, 22, 23). The fact that even on post-contrast studies BME was noted on acute fracture may be advantageous, as the majority of CTs in the trauma setting are performed with iodinated contrast given the need to evaluate vascular or solid organ injuries. Additional research needs to be done to better understand the role of iodinated contrast in cervical and thoracolumbar BME evaluation on DECT imaging.

### Limitations

The limitations for this study include its retrospective design. As an inclusion criterion, we only assessed fractures that had MRI correlate within 3 weeks of the DECT to make appropriate measurements. These criteria significantly narrowed the types of fractures that were included in this cohort. For example, certain types of posterior element fractures were not collected because these do not always mandate MRI evaluation.

A second limitation is the small sample size. Although the differences in this study were statistically significant, a larger sample size may increase the statistical power.

## Conclusions

Two-material decomposition GSI DECT can demonstrate quantitatively significant BME in the emergency setting, possibly allowing for an improved detection of acute thoracolumbar vertebral fractures. In contradistinction, DECT was unable to reliably detect a bone marrow edema in cervical spinal fractures.

## Data Availability

The original contributions presented in the study are included in the article/Supplementary Material, further inquiries can be directed to the corresponding author.
